# Resolutions on the Death of Dr. J. J. R. Patrick

**Published:** 1895-08

**Authors:** 


### Obituary.

### RESOLUTIONS ON THE DEATH OF DR. J. J. R. PATRICK.

Resolutions adopted by the Odontological Society of Chicago in reference to the death of Dr. J. J. R. Patrick, of Belleville, Ill.:

Whereas, Death has removed from our midst Dr. John J. R. Patrick, of Belleville, Illinois, and there has passed from the scenes of human activity a character whose labors have enriched the stores of science, a man who delved fearlessly and steadfastly into those mysteries of nature which call forth the most subtle energies of the human mind, and yield results making the lives of succeeding generations happier; now, therefore, be it

*Resolved*, That, in the death of Dr. Patrick the world loses an honored citizen-soldier, the field of science a conscientious, faithful laborer, and the dental profession a light whose extinguishment will kindle the profession's interest in the work he has accomplished for it, and thus extend and perpetuate his good influence. Be it further

*Resolved*, That to the family, friends, and professional associates of Dr. Patrick we extend the assurance that we appreciate his worth, and are grateful that his life had been spared until it nearly completed the allotted time of three-score and ten.

James A. Swasey,
Alison W. Harlan,
Louis Ottofy,
*Committee.*

